# Supplementary figures and images for: Humoral immune response to adenovirus induce tolerogenic bystander dendritic cells that promote generation of regulatory T cells
Source: PLoS Pathog. 2018 Aug 20;14(8):e1007127. doi: 10.1371/journal.ppat.1007127 (PMC6117092; doi:10.1371/journal.ppat.1007127)

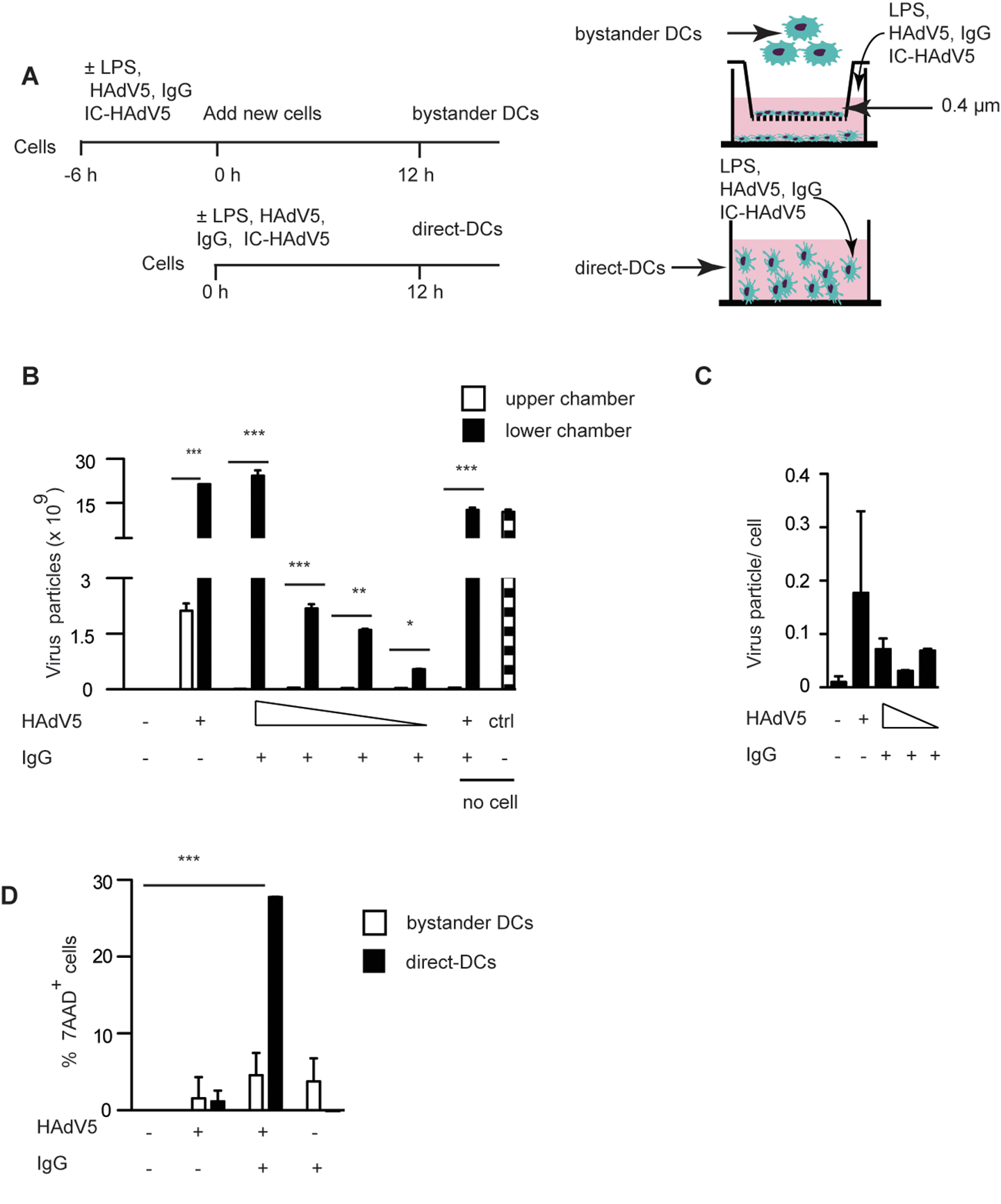

Supplement: S1 Fig — A) We used transwell inserts with 0.4 um filter to generate direct and bystander DCs. Direct DCs (1.5 x10^6^ cells unless mentioned otherwise) were incubated with the stimulus (e.g. LPS, HAdV5, mutant virus, ± IVIg ± drugs) in lower compartment for 6 h. Fresh DCs (6 x 10^5^) were added to the upper compartment. B) To determine if HAdV5 particles (2 x 10^4^ pp/ml) added to the lower chamber diffused to the upper compartment and impact the bystander DCs, we quantified (by qPCR) HAdV5 genomes in the supernatant of each compartment. 1.6 x 10^10^ pp of HAdV5 pp were used in the control medium. These data demonstrate that 10,000-fold fewer particles could be found in the upper chamber. C) Quantification of HAdV5 genomes associated with bystander DCs as measured by qPCR (n ≥3). DNA from mock-treated samples was extracted and virus/cell was normalized to GAPDH copy number. The quantity of HAdV5 genomes/cell was normalized by lacZ (transgene in the vector) vs. GAPDH copy number. While direct DCs take up ~600 pp/cell [14], we found that 1 in 10 bystander DC contains a single HAdV5 genome. D) The 7AAD+ bystander and direct DCs (i.e. DCs with compromised plasma membrane integrity) in each condition were quantified by flow cytometry. The assays were carried out in 4 donors (mean ± SEM. These results demonstrate that bystander DCs do not show loss of cell membrane integrity. p values were derived using Student’s t-test (B & C) or one-way ANOVA with Dunnett’s post-tests (D). * p < 0.05, ** p < 0.01 and *** p < 0.001. (TIF) [file ppat.1007127.s001.tif]

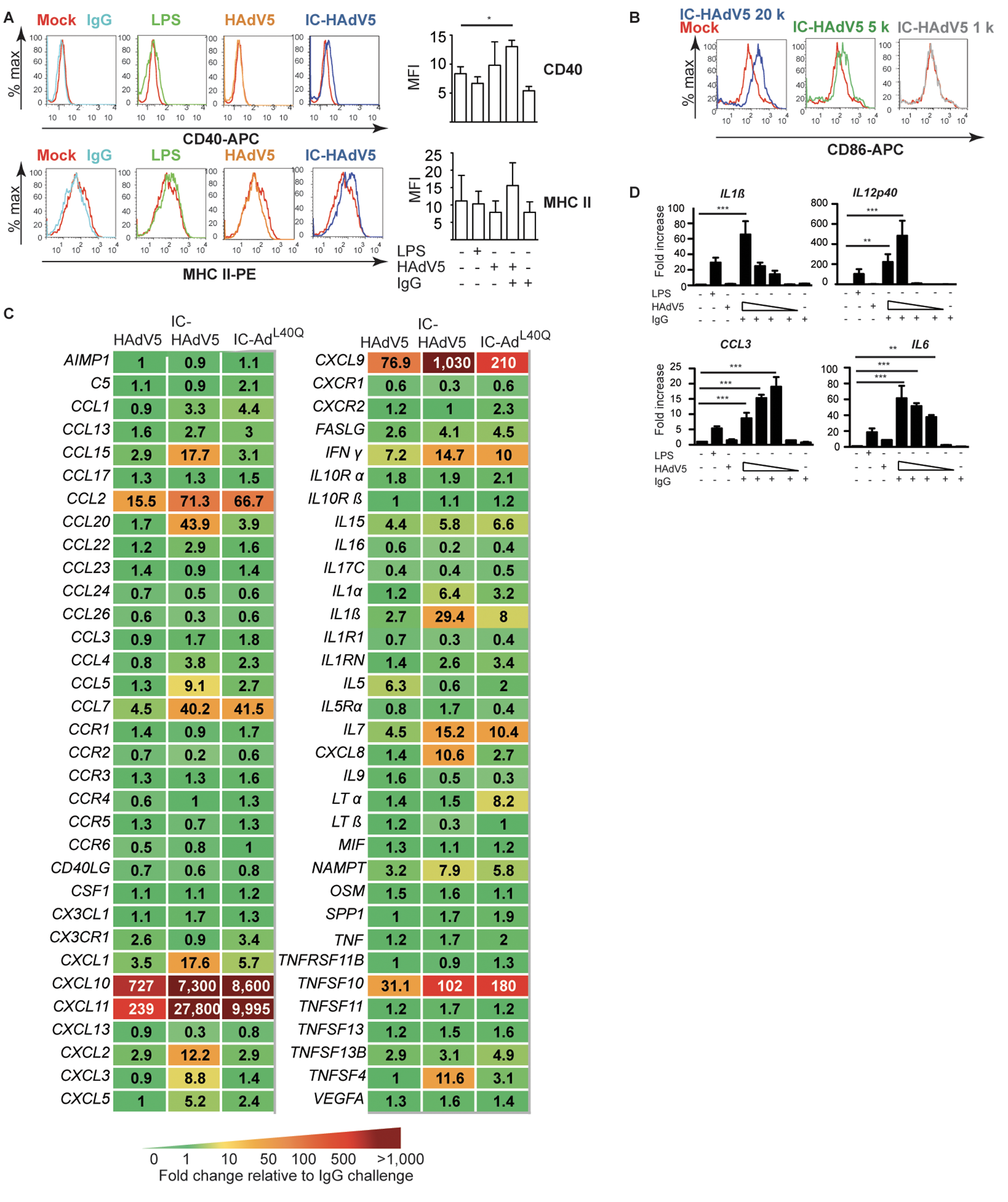

Supplement: S2 Fig — Bystander DCs were generated using milieu from DCs challenged with IgG, LPS, HAdV5, or IC-HAdV5. The color code is as in Fig 2. A) The data are representative flow cytometry profiles of CD40 and MHC II surface expression. A modest increase was noted in each case. B) In a dose-dependent assay (20,000, 5,000, or 1,000 pp/cell) CD86 cell surface levels were quantified detected by flow cytometry. The data are representative flow cytometry profiles. Assays were carried out in 4 donors with similar results. C) PCR array profiles from bystander DCs exposed to the milieu generated by DCs challenged by HAdV5, IC-HAdV5, and IC-AdL40Q. The 66 cytokine mRNAs that gave unique qPCR peaks in our hands. D) IL1β, IL12p40, CLL3 and IL6 mRNA levels in bystander THP1 DCs assayed in a dose-dependent (20,000, 10,000, 5,000, or 1,000 pp/direct DC) response. Data are mean ± SEM with 3 independent experiments. p values were derived from one-way ANOVA with Dunnett’s test. * p < 0.05, ** p < 0.01 and *** p < 0.001. (TIF) [file ppat.1007127.s002.tif]

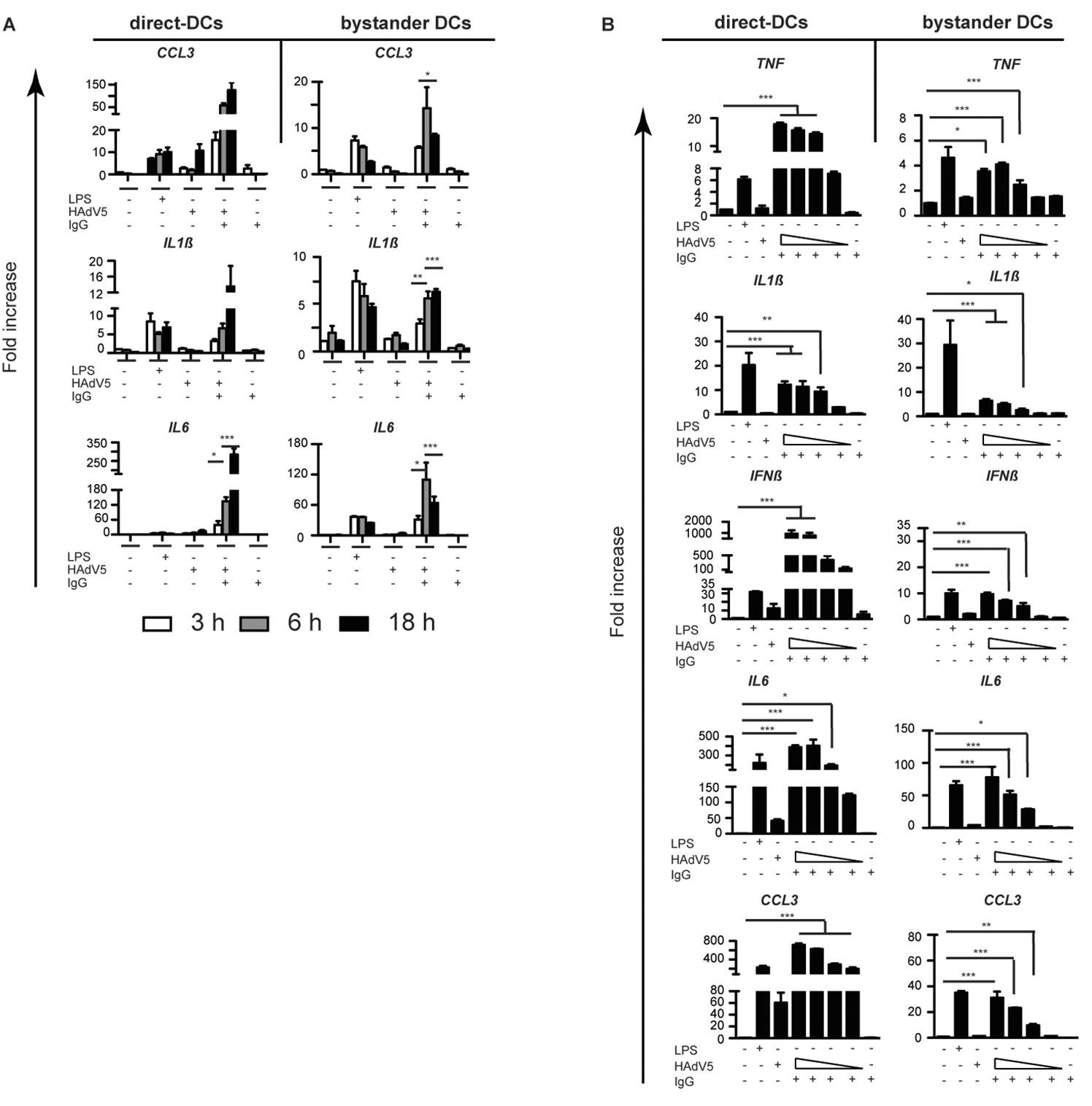

Supplement: S3 Fig — We extended the mRNA array results by quantifying dose-dependent responses of a handful of mRNA levels by RT-qPCR. Because DCs derived from monocytes from random blood bank donors can have widely different levels of mRNAs, we compared mRNA levels in THP-1-derived DCs to provide a standardized view of the changes. THP-1 cells were differentiated into DCs for 6 days, then directly and indirectly activated. A) CCL3, IL1β, and IL6 mRNA levels in DCs challenged with LPS, IgG, HAdV5 and IC-HAdV5 (left hand column), and bystander DCs (right hand column) incubated in the respective direct DC milieu were quantified at 3, 6, and 18 h post-incubation. B) Changes in TNF, IL1β, IFNβ, IL6, and CCL3 mRNA levels in direct (left hand column) TNF: IC 2 x 10^4^ vs. 104 ns; 104 vs. 5 x 10^3^ ns; 5 x 10^3^ vs. x 103 ***; IL1β IC 2 x 10^4^ vs. 104 ns; 104 vs. 5 x 10^3^ ns; 5 x 10^3^ vs. 103 ns; IC 2 x 10^4^ vs. 103 **, IC 104 vs. 103 *; IFNβ: IC 2 x 10^4^ vs. 104 ns; 104 vs. 5 x 10^3^ ns; 5 x 10^3^ vs. 103 ns, IC 2 x 10^4^ vs. 103 *; IL6: IC 2 x 10^4^ vs. 104 ns; 104 vs. 5 x 10^3^ **; 5 x 10^3^ vs. 1 x 10^3^ ns; CCL3: IC 2 x 10^4^ vs. 1 x 10^4^ ns; 1 x 10^4^ vs. 5 x 10^3^ ***; 5 x 10^3^ vs. 103 ns) Bystander DC (right hand column) dose-dependent assay (2 x 10^4^, 104, 5 x 10^3^, or 103 pp/cell) by RT-qPCR TNF: IC 2 x 10^4^ vs. 104 ns; 104 vs. 5 x 10^3^ ns; 5 x 10^3^ vs. 1 k ns, IC 2 x 10^4^ vs. 103 **, IC 104 vs. 103 ***; IL1β: IC20 k vs. 104 ns; 104 vs. 5 x 10^3^ ns; 5 x 10^3^ vs. 103 ns; IFNβ: IC 20 k vs. 104 ns; 104 vs. 5 x 10^3^ ns; 5 x 10^3^ vs. 103 **; IL6: IC 2 x 10^4^ vs. 104 ns; 104 vs. 5 x 10^3^ ns; 5 x 10^3^ vs. 103 ns, 104 vs. 5 x 10^3^ ***; CCL3: IC 2 x 10^4^ vs. 104 ns; 104 vs. 5 x 10^3^ ***; 5 x 10^3^ vs. 103 *). As in “A” controls included IgG and HAdV5. Three independent experiments were carried out. Data are mean ± SEM. p values were derived using Student’s t-tests. * p < 0.05, ** p < 0.01 and *** p < 0.001. (TIF) [file ppat.1007127.s003.tif]

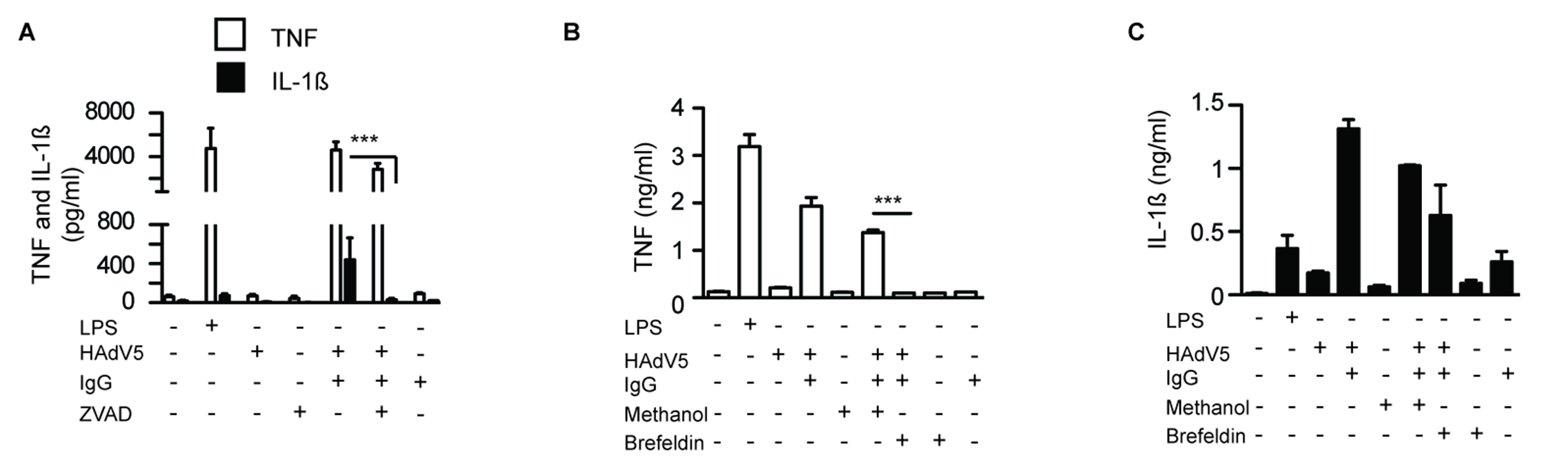

Supplement: S4 Fig — A) TNF and IL-1β secretion in response to ZVAD treatment (2 h before challenge) of DCs challenged with LPS, IgG, HAdV5, and IC-HAdV5. B) DCs were simultaneously treated with brefeldin A and challenged with LPS, IgG, HAdV5, and IC-HAdV5. TNF secretion was quantified at 18 h. C) DCs were simultaneously treated with brefeldin A and challenged with LPS, IgG, HAdV5, and IC-HAdV5. IL-1β secretion was quantified at 18 h. Data are mean ± SEM, p values were derived from Student’s t-tests, n ≥ 3 donors. *** p < 0.001. (TIF) [file ppat.1007127.s004.tif]

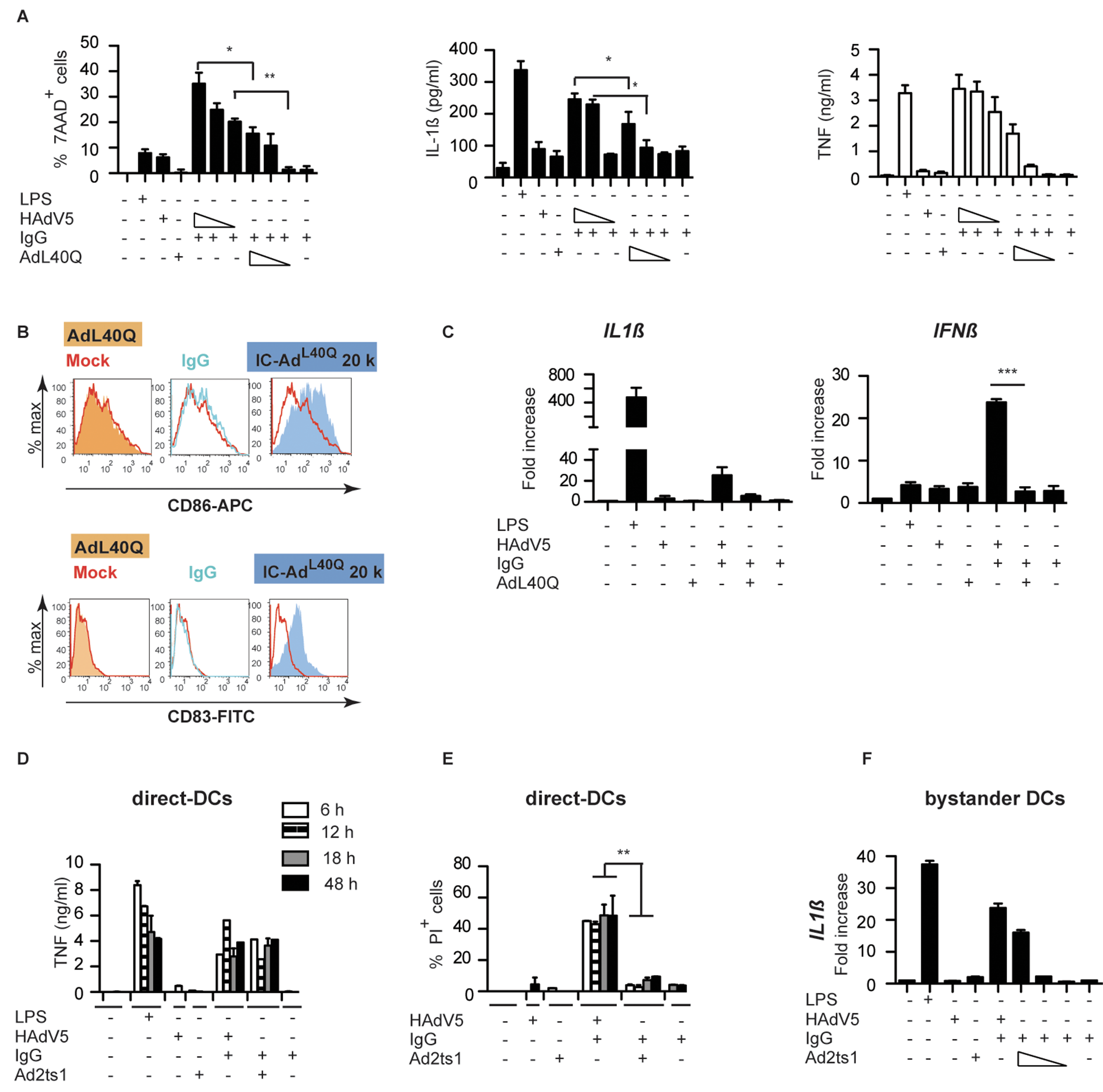

Supplement: S5 Fig — A) DCs challenged with LPS, IgG, HAdV5, AdL40Q and increasing concentrations of IC-HAdV5 and IC-AdL40Q were analyzed for loss of membrane integrity (7AAD+ cells), IL-1β and TNF secretion. B) Cell surface levels of the maturation/activation markers CD86 and CD83 following direct DCs challenged with IgG, AdL40Q, IC-AdL40Q, HAdV5, and IC-HAdV5. C) bystander DC IL1β and IFNβ mRNA levels quantified by RT-qPCR assay. Experiments were carried out in ≥3 donors. p values were derived from Student’s t-tests. *, **, *** denote p values of < 0.05, < 0.01, < 0.001, respectively. DCs were challenged with LPS, IgG, HAdV5, IC-HAdV5, Ad2ts1, and IC-Ad2ts1 and screened for D) time-dependent (6 to 48 h) TNF secretion; and E) time-dependent (6 to 48 h) loss of membrane integrity using propidium iodide (PI) incorporation; or F) DCs were challenged with LPS, IgG, HAdV5, IC-HAdV5, Ad2ts1, and IC-Ad2ts1 and then used to generate bystander DCs in which the IL1β mRNA levels were quantified by RT-qPCR assay following dose-dependent stimulation (20 x 103, 10 x 103, or 5 x 10^3^ pp/cell) of the direct DCs. Experiments were carried out in 3 donors and in duplicate. P values were derived from Student’s t-tests. ** p < 0.01. (TIF) [file ppat.1007127.s005.tif]

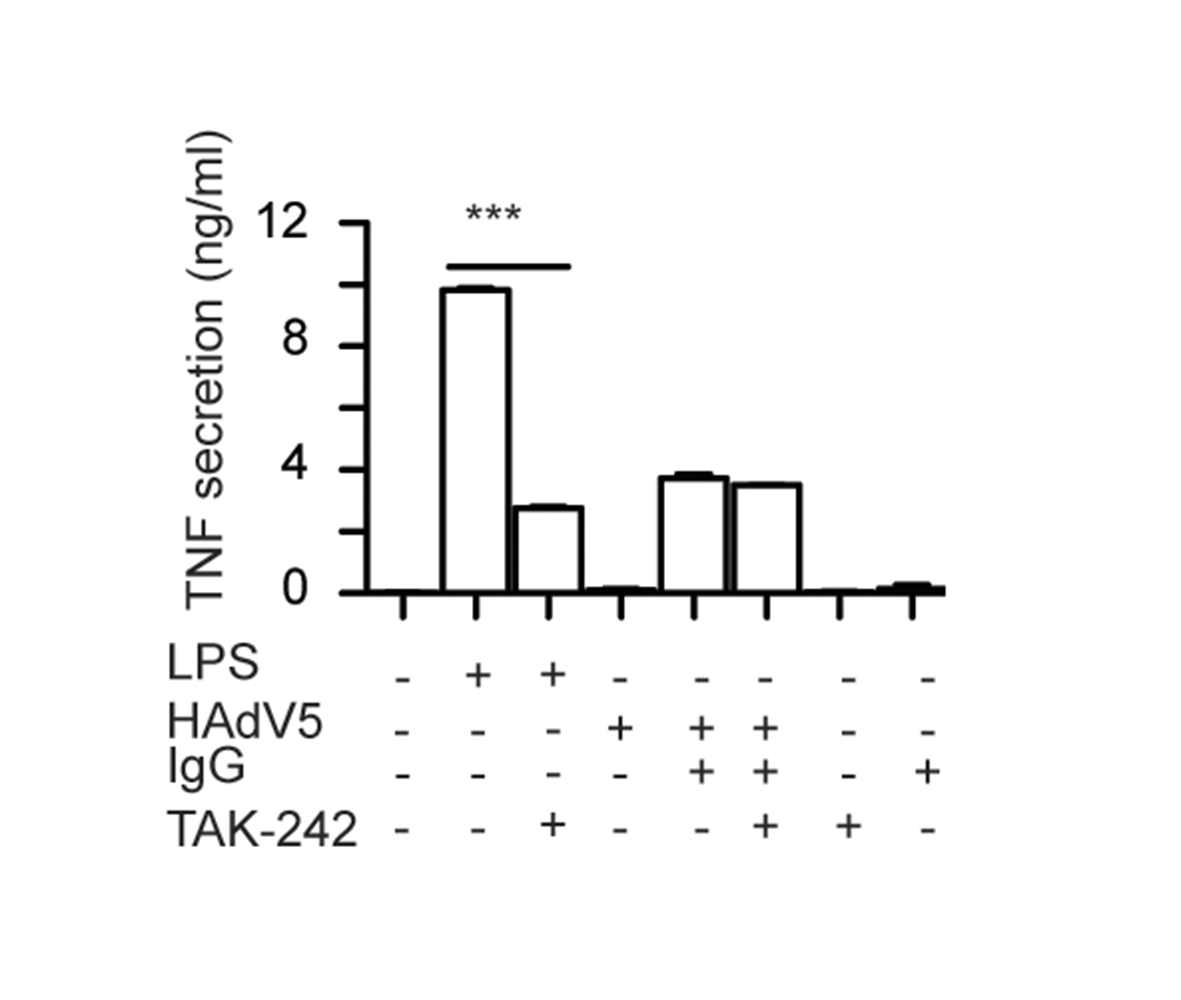

Supplement: S6 Fig — Bystander DCs were treated with TAK-242 for 1 h before adding them to the DCs challenged with LPS, IgG, HAdV5, or IC-HAdV5. TNF secretion was quantified in direct DCs in the lower compartment (n = 3 donors). p values were derived from Student’s t-tests. *** p < 0.0001. (TIF) [file ppat.1007127.s006.tif]

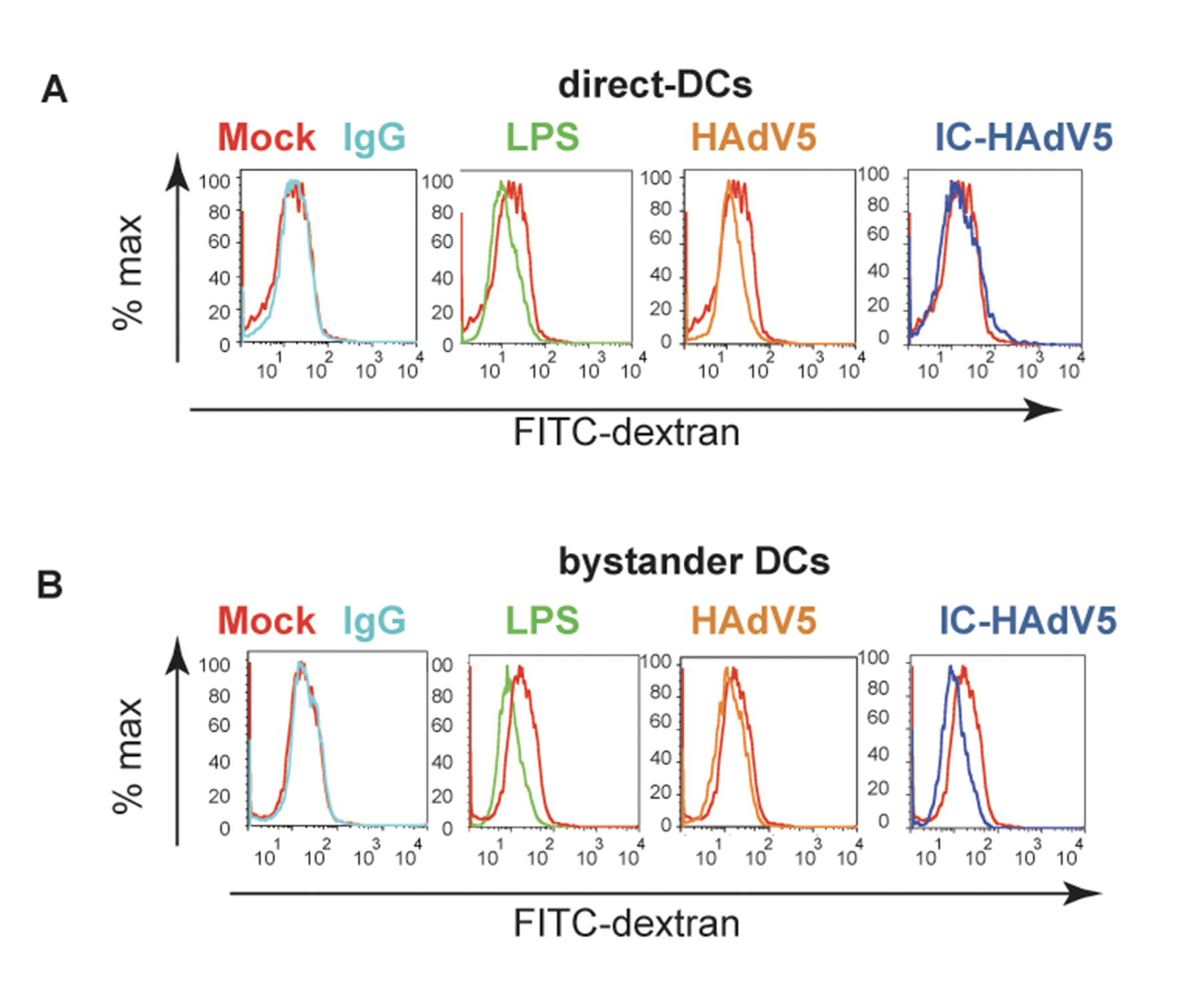

Supplement: S7 Fig — Nonspecific binding of dextran to A) direct DCs and B) bystander DCs was controlled by incubating DC (post-stimulation) with FITC-labeled dextran at 4°C. Direct DCs were challenged with IgG, LPS, HAdV5, or IC-HAdV5. The cells were then incubated with FITC-labeled dextran and analyzed by flow cytometry. The data are representative flow cytometry profiles with experiments performed using cells from in 3 donors and in duplicate. (TIF) [file ppat.1007127.s007.tif]

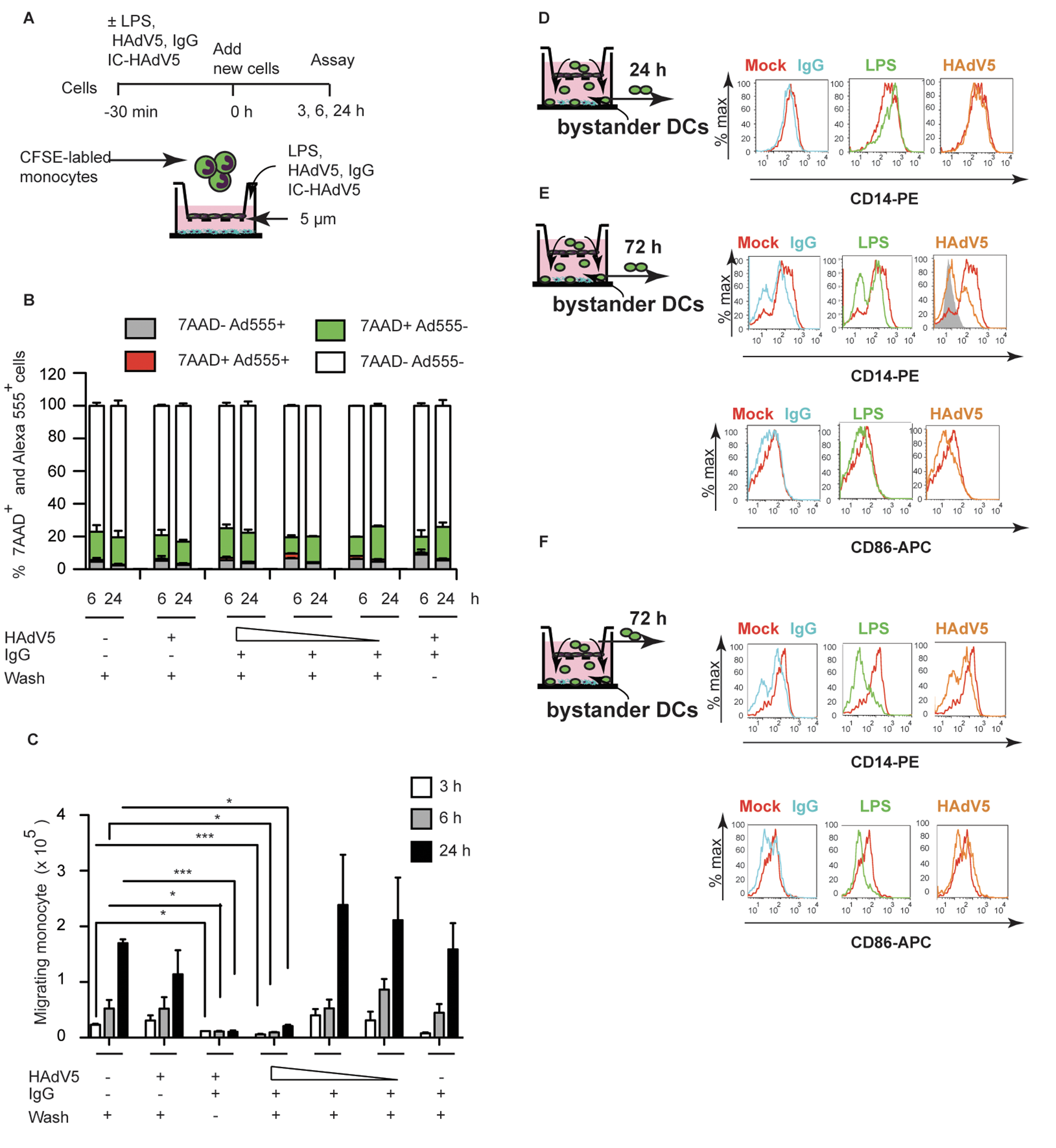

Supplement: S8 Fig — A) A 5-micron-pore membrane transwell system was used for monocyte migration assays. The timing and stimuli are indicated in the schematics. Round green cells are CFSE-labeled monocytes. B) These data shown percentage of monocyte in the upper chamber that potentially interact with HAdV or IC-HAdV5. C) To address this possibility, we covalently linked Alex555 to the HAdV5 capsid (HAdV5-Alexa555 [29]) to identify cells associated with HAdV5 or IC-HAdV5. CFSE-labeled monocytes were then assayed by flow cytometry for loss of membrane integrity (7AAD+ cells) and the presence of HAdV5-Alexa555 at 6 and 24 h. These data demonstrate that ICs do not go through the pore to interact with monocytes in the upper chamber. D) CD14 expression levels on monocytes recruited towards bystander DCs that were created with the milieu from DCs challenged with IgG, LPS or HAdV5 at 24 h. E) CD14 and CD86 levels on monocytes recruited to bystander DCs that were created with the milieu from DCs challenged with IgG, LPS or HAdV5 at 72 h. F) CD14 and CD86 expression levels on monocytes that remained in the upper compartment at 72 h. The lower compartment contained bystander DCs that were created with the milieu from DCs challenged with IgG, LPS or HAdV5. The data are representative flow cytometry profiles with assays carried out in 4 donors. * p < 0.05, ** p < 0.01 and *** p < 0.001. (TIF) [file ppat.1007127.s008.tif]

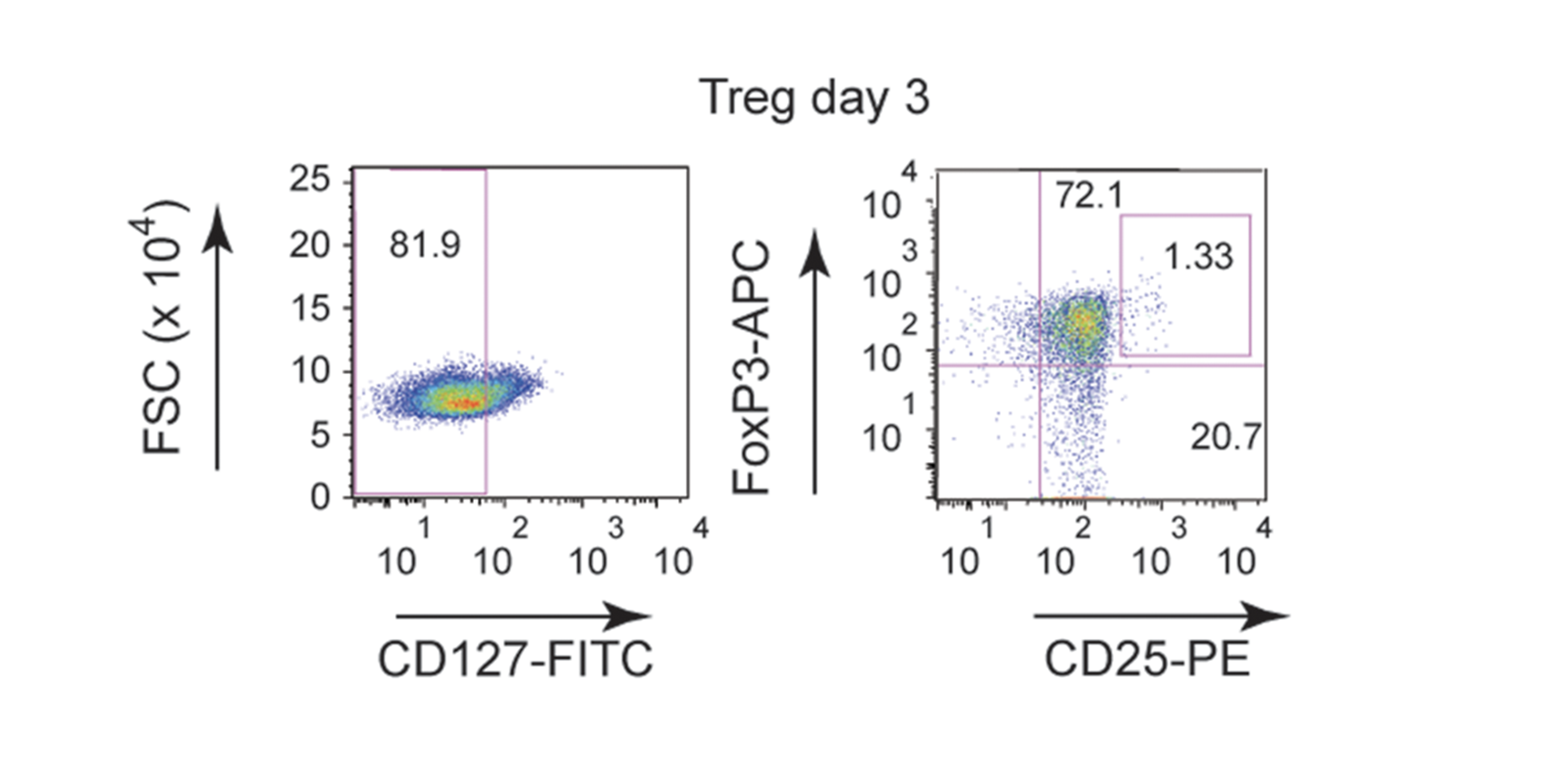

Supplement: S9 Fig — Three days post-incubation we gated on CD127dim cells to identify CD25+/FoxP3high cells. The data are representative flow cytometry profiles with assays carried out in 7 donors. (TIF) [file ppat.1007127.s009.tif]

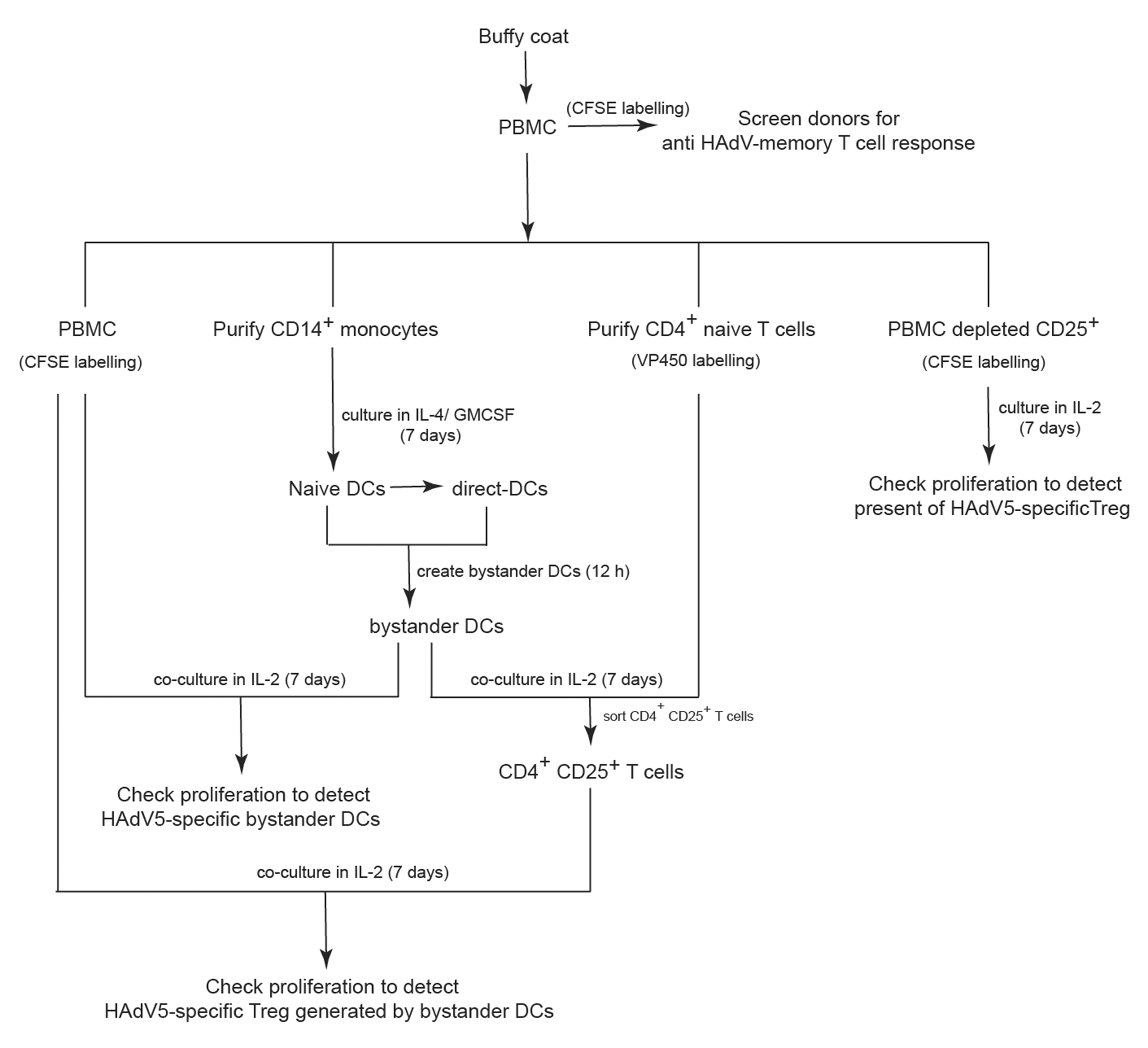

Supplement: S10 Fig — (TIF) [file ppat.1007127.s010.tif]

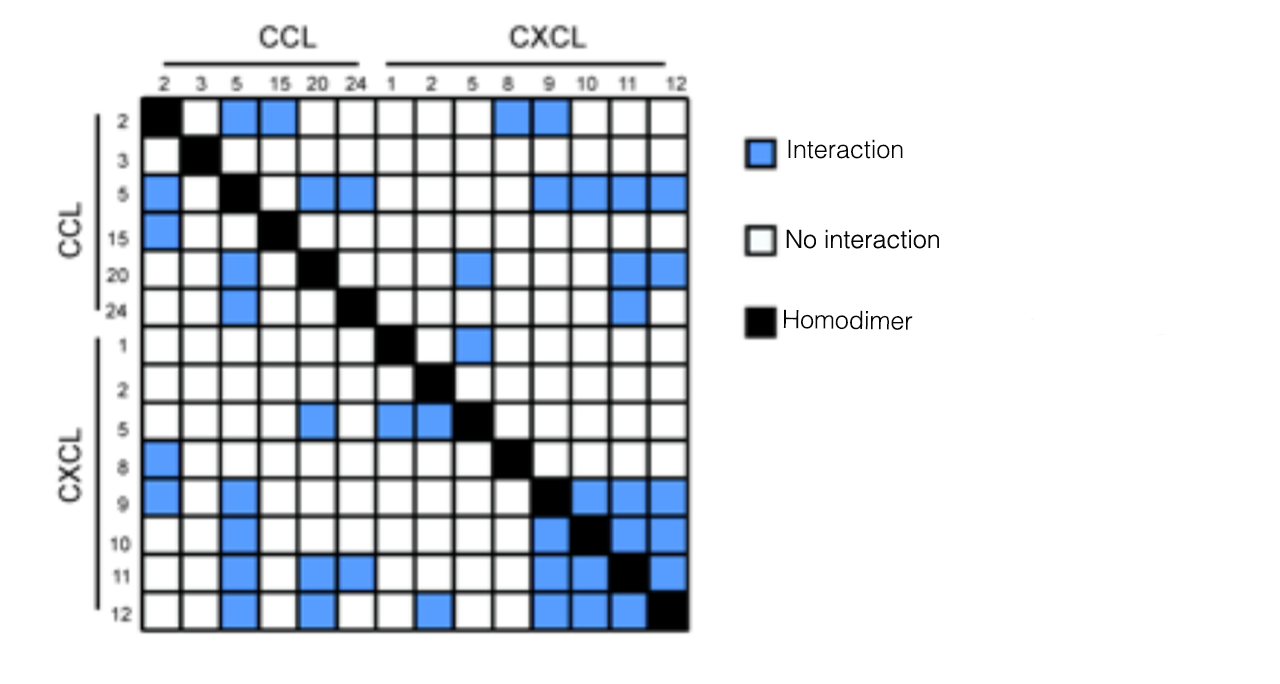

Supplement: S11 Fig — Potential cytokine heterodimers are based on von Hundelshausen et al. [56] interactome data and the response generated by direct and bystander DCs. (TIF) [file ppat.1007127.s011.tif]
