# Supplementary material for: Humoral immune response to adenovirus induce tolerogenic bystander dendritic cells that promote generation of regulatory T cells
Source: PLoS Pathog. 2018 Aug 20;14(8):e1007127. doi: 10.1371/journal.ppat.1007127 (PMC6117092; doi:10.1371/journal.ppat.1007127)
Supplement: S1 Table — (DOCX) [file ppat.1007127.s012.docx]

**S1 Table) Statistical analyses of bystander DC cytokine transcription profile**

| **cytokine** | **condition 1** | **condition 2** | **significance** |
| --- | --- | --- | --- |
| ***TNF*** | IC 2 x 10^4^ pp/cell | IC 10^4^ pp/cell | p <0.0001 |
|  | IC 1 x 10^4^ pp/cell | IC 5 x10^3^ pp/cell | p >0.05 |
|  | IC 5 x 10^3^ pp/cell | IC 1 x 10^3^ pp/cell | p <0.0001 |
| ***IFNβ*** | IC 2 x 10^4^ pp/cell | IC 1 x 10^4^ pp/cell | p <0.0001 |
|  | IC 1 x 10^4^ pp/cell | IC 5 x 10^3^ pp/cell | p >0.05 |
|  | IC 5 x 10^3^ pp/cell | IC 1 x 10^3^ pp/cell | p <0.0001 |
| ***CXCL10*** | IC 2 x 10^4^ pp/cell | IC 1 x 10^4^ pp/cell | p <0.0001 |
|  | IC 1 x 10^4^ pp/cell | IC 5 x 10^3^ pp/cell | p >0.05 |
|  | IC 5 x 10^3^ pp/cell | IC 1 x 10^3^ pp/cell | p <0.0001 |
