# Supplementary material for: Humoral immune response to adenovirus induce tolerogenic bystander dendritic cells that promote generation of regulatory T cells
Source: PLoS Pathog. 2018 Aug 20;14(8):e1007127. doi: 10.1371/journal.ppat.1007127 (PMC6117092; doi:10.1371/journal.ppat.1007127)
Supplement: S2 Table — (DOCX) [file ppat.1007127.s013.docx]

**S2 Table) In-house designed primer sequences**

| **Gene** | **Primer sequences** |
| --- | --- |
| *TNF* | 5’- CTCTGGCCCAGGCAGTCAGA -3’ [forward] |
|  | 5’- GGCGTTTGGGAAGGTTGGAT -3’ [reverse] |
| *IL1β* | 5’- AAACAGATGAAGTGCTCCTTCC -3’ [forward] |
|  | 5’- AAGATGAAGGGAAAGAAGGTGC -3’ [reverse] |
| *IFNβ* | 5’- GTCTCCTCCAAATTGCTCTC -3’ [forward] |
|  | 5’- ACAGGAGCTTCTGACACTGA -3’ [reverse] |
| *CXCL10* | 5’- TATTCCTGCAAGCCAATTTTGTC -3’ [forward] |
|  | 5’- TCTTGATGGCCTTCGATTCTG -3’ [reverse] |
| *IL6* | 5’- CCAGGAGCCCAGCTATGAAC -3’ [forward] |
|  | 5’- CCCAGGGAGAAGGCAACTG -3’ [reverse] |
| *IL12 (p40)* | 5’- CCAAGAACTTGCAGCTGAAG -3’ [forward] |
|  | 5’- TGGGTCTATTCCGTTGTGTC -3’ [reverse] |
| *CCL3* | 5’- CTGCATCACTTGCTGCTGACA -3’ [forward] |
|  | 5’- CACTGGCTGCTCGTCTCAAAG -3’ [reverse] |
| *GAPDH* | 5’-ACAGTCCATGCCATCACTGCC-3’ [forward] |
|  | 5’-GCCTGCTTCACCACCTTCTTG-3’ [reverse] |
